# Bovine Coronavirus: Variability, Evolution, and Dispersal Patterns of a No Longer Neglected Betacoronavirus

**DOI:** 10.3390/v12111285

**Published:** 2020-11-10

**Authors:** Giovanni Franzo, Michele Drigo, Matteo Legnardi, Laura Grassi, Daniela Pasotto, Maria Luisa Menandro, Mattia Cecchinato, Claudia Maria Tucciarone

**Affiliations:** Department of Animal Medicine, Production and Health (MAPS), University of Padua, 35020 Legnaro, Italy; michele.drigo@unipd.it (M.D.); matteo.legnardi@phd.unipd.it (M.L.); laura.grassi.2@phd.unipd.it (L.G.); daniela.pasotto@unipd.it (D.P.); marialuisa.menandro@unipd.it (M.L.M.); mattia.cecchinato@unipd.it (M.C.); claudiamaria.tucciarone@unipd.it (C.M.T.)

**Keywords:** bovine coronavirus, phylodynamics, phylogeography, host, selection, evolution

## Abstract

Bovine coronavirus (BoCV) is an important pathogen of cattle, causing severe enteric disease and playing a role in the bovine respiratory disease complex. Similar to other coronaviruses, a remarkable variability characterizes both its genome and biology. Despite their potential relevance, different aspects of the evolution of BoCV remain elusive. The present study reconstructs the history and evolution of BoCV using a phylodynamic approach based on complete genome and spike protein sequences. The results demonstrate high mutation and recombination rates affecting different parts of the viral genome. In the spike gene, this variability undergoes significant selective pressures—particularly episodic pressure—located mainly on the protein surface, suggesting an immune-induced selective pressure. The occurrence of compensatory mutations was also identified. On the contrary, no strong evidence in favor of host and/or tissue tropism affecting viral evolution has been proven. The well-known plasticity is thus ascribable to the innate broad viral tropism rather than mid- or long-term adaptation. The evaluation of the geographic spreading pattern clearly evidenced two clusters: a European cluster and an American–Asian cluster. While a relatively dense and quick migration network was identified in the former, the latter was dominated by the primary role of the United States (US) as a viral exportation source. Since the viral spreading pattern strongly mirrored the cattle trade, the need for more intense monitoring and preventive measures cannot be underestimated as well as the need to enforce the vaccination of young animals before international trade, to reduce not only the clinical impact but also the transferal and mixing of BoCV strains.

## 1. Introduction

Bovine coronavirus (BoCV) belongs to the family *Coronaviridae*, genus *Betacoronavirus* (https://talk.ictvonline.org/). The genome is a single-stranded positive-sense RNA of about 31 Kb, which includes ten open reading frames (ORFs) flanked by 5′ and 3′ untranslated regions [1]. ORF1 codes for the polyproteins pp1a and—through a ribosomal frameshift—for pp1ab, which are then proteolytically cleaved into multiple non-structural proteins (NSP) [2]. ORF3, 4, 8, 9, and 10 code for the structural proteins hemagglutinin–esterase protein (HE), spike glycoprotein (S), small membrane protein (SE), membrane protein (M), and nucleocapsid protein (N). Other ORFs encode additional NSPs, such as 32 kDa and 12 kDa, whose functions have been less characterized [1]. As for other coronaviruses, the S protein is the most studied, constituting the typical protrusions on the viral surface. It is cleaved into two subunits: S1 and S2. S1 is involved in viral attachment and is thus a major determinant of host and tissue tropism. Additionally, it is considered the main target of the host immune response and several epitopes, including neutralizing ones, have been identified. S2 is involved in membrane fusion [3,4] and contains the transmembrane domains anchoring the glycoprotein to the viral envelope.

Although the genetic diversity of BoCV appears to be low compared to other members of this family, coronaviruses belong to the group of rapidly evolving viruses, featured by the continuous generation of new variants [5]. This plasticity can provide an evolutionary advantage in rapidly changing environments, allowing adaptation to different tissues and hosts, the escape of the immune response, and even allowing BoCV to cope with animal populations with different structures and rearing conditions. BoCV was first recognized as an enteric pathogen that is involved in calf diarrhea (NCD) and winter dysentery (WD) in adult cattle. It was then associated with respiratory signs in animals of all ages, and, in the presence of other viruses and bacteria, it takes part in the bovine respiratory disease complex (BRDC) [6,7]. Despite the different studies and epidemiological investigations that have been performed over time, the issue of enteric/respiratory signs possibly being linked to specific strains/mutations is still debated [4,8].

Currently, no clear phylogenetic clustering has been reported among strains causing different symptomatology. On the contrary, even if the infection is distributed worldwide, a certain geographical separation has been identified, distinguishing a European type from an American–Asian type [1,9].

Besides cattle, different local domestic and wild ruminants, including several species of deer, waterbuck antelope, giraffe, alpaca, sable antelope, and others (see Amer et al. for review [10]), were reported to be susceptible to BoCV infection, even in the presence of overt clinical signs. Overall, BoCV causes relevant direct economic losses due to mortality, reduced growth, and milk production, in addition to the indirect losses due to a more challenging animal management [4,11]. The increased use of antimicrobials also presents a critical concern because of the limitations imposed by the legislation, the consumers’ opinion and requests, especially in the European Union [12,13]. In spite of several reports, no dedicated attempt has yet been made to globally evaluate the pattern of BoCV spread among countries and hosts and how the viral population has changed and evolved over time.

The present study attempts to fill this gap by performing an extensive evolution analysis based on all strains whose complete genome was available. Additionally, because of its biological relevance and to maximize the sample size and available information, a dedicated and more extensive phylodynamic analysis has been performed based on viral spike sequences.

## 2. Materials and Methods

### 2.1. Compete Genome Dataset and Recombination Analysis

All available BoCV complete genomes were downloaded from GenBank. The sequences were preliminarily aligned using MAFFT [14], and the alignment was visually inspected to identify and remove poorly aligned regions or low-quality sequences. Recombination analysis was performed using RDP4 [15], selecting RDP, GENECONV, Chimaera, and 3Seq for the primary scan, while the whole set of available methods was used for recombination confirmation. Each method’s settings were adjusted based on the dataset features according to the RDP4 manual. Recombination events were accepted only if detected by more than two methods with a significance level of *p* < 0.001 with Bonferroni correction. Recombination breakpoint density plots were constructed using the same program. Similar to the approach followed by Lefeuvre et al. [16], the differences in breakpoint densities were also tested between:

1. Coding and non-coding regions;
2. Each ORF and the remainder of the genome;
3. Between the ends of the coding regions (external 25%) and the middle of these regions.

To further confirm the occurrence of recombination, a phylogenetic network was reconstructed using the NeighborNet method based on the whole genome sequences using SplitsTree4 [17].

### 2.2. Gene Evolution Rate

Complete genomes of viral strains provided with collection dates were divided into different ORFs. The obtained sequences were aligned at the codon level using TranslatorX [18]. Gene-specific datasets were scanned for recombinant identification as previously described, and recombinant sequences were removed from the dataset. The absence of residual recombination events was assessed using GARD [19]. A preliminary maximum likelihood (ML) phylogenetic tree was reconstructed using Iq-Tree [20] and used to evaluate the presence of an adequate temporal signal using TempEst v1.5 [21] to perform a regression of the root-to-tip distances of the ML tree and sampling dates. The evolutionary rate, as well as other parameters of interest (i.e., the time to most recent common ancestor (tMRCA) and population dynamics), were jointly estimated using the Bayesian serial coalescent approach implemented in BEAST 1.10 [22]. The substitution model was selected based on the Bayesian Information Criterion (BIC) calculated using Jmodeltest [23], while the relaxed lognormal molecular clock was preferred over the strict one based on the Bayesian Factor (BF) (i.e., BF = 11.23) calculation through marginal likelihood estimation performed using Stepping Stone (SS) and Path Sampling (PS) approaches [24]. The non-parametric Skygrid model was selected to reconstruct and account for the variation in the viral population size over time [25]. The parameters and tree were estimated using a 100 million-generation-long Markov Chain Monte Carlo (MCMC) run, sampling every one thousand states. The results of the run were accepted only if the estimated sample size was bigger than 200 and the mixing and convergence of the runs, visually evaluated using Tracer, were adequate. The posterior distribution was summarized in terms of the mean, median, and 95% high posterior density (95HPD) after the exclusion of a burn-in equal to 20% of the run length. Maximum clade credibility (MCC) trees were constructed and annotated using Treeannotator (BEAST package).

### 2.3. Spike Dataset

A collection of all complete S sequences of strains for which the collection date, country, and host were available were downloaded from GenBank. Sequences were aligned at the codon level, and the quality of the dataset and absence of recombination were evaluated as previously described. However, in this case, the spreading pattern of BoCV among countries was reconstructed over time using the discrete phylogeographic approach described by Lemey et al. [26]. The Bayesian Stochastic Search Variable Selection (BSSVS) was also implemented, allowing the identification of the most parsimonious description of the spreading process and thus the well-supported migration rates among countries through BF calculation. The same approach was selected to reconstruct and evaluate the transmission process among host species. MCMC run results were evaluated, and population parameters and trees were summarized as previously described. SpreaD3 [27] was used to calculate the BF, indicative of statistically supported country/host transition events, and plot viral spreading over time and space. Other figures were plotted using the ggplot2 library in R [28]. For all analyses, a BF >10 was considered significant. The presence and strength of pervasive diversifying selective pressures acting on each site of the S protein were investigated using the SLAC, FEL, and FUBAR methods [29,30], while the presence of episodic diversifying selection was assessed using MEME [31]. The occurrence of viral lineages under this type of selection was evaluated using aBSREL [32]. The Bayesian Graphical Model (BGM) was used to identify co-evolving sites [33,34]. Finally, the presence of amino acids under directional episodic selection in respiratory strains compared to enteric ones was tested using MEDS [35], setting the tree branches leading to respiratory strains as the foreground. All mentioned methods were implemented in HyPhy [36]. The significance level was set to *p* < 0.05 or a posterior probability of >0.9.

### 2.4. Homology Modelling

The S gene nucleotide sequence of the representative strain U00735.2 was translated at the amino acid level, and the SWISS-MODEL web server [37] was used to identify the best template for which a quaternary structure had been experimentally determined, and the protein structure was estimated through a homology-modeling approach. The obtained model was visualized and edited with Chimera [38].

## 3. Results

### 3.1. Complete Genomes

#### 3.1.1. Recombination Rate Analysis

**Figure 1:**
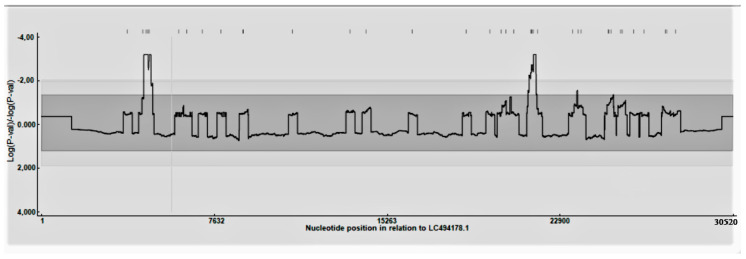
Recombination distribution plot. The significance (*p*-value) of hot and cold-recombination spots is reported along the genome.

A total of 111 complete genomes were included in the analysis. The 5′ and 3′ untranslated regions (UTRs) were excluded to deal with the variable completeness of the available genomes. According to the defined parameters, most of the sequences (i.e., 57) had recombination evidence. No significant differences were observed in recombination frequency occurrence among ORFs or different regions of the ORFs and/or intergenic regions. However, the breakpoint distribution plot evidenced the presence of some recombination hot-spots located around positions 4700 (pp1a), 21,600 (end of pp1ab/junction with region 32 KDa protein), and 23,900 (beginning of S) (based on the Mebus strain coding region) (Figure 1). The results were confirmed by NeighborNet reconstruction, demonstrating the presence of several reticulations, and by the Phy test, which found significant evidence of recombination.

#### 3.1.2. Gene Evolutionary Rate

**Figure 2:**
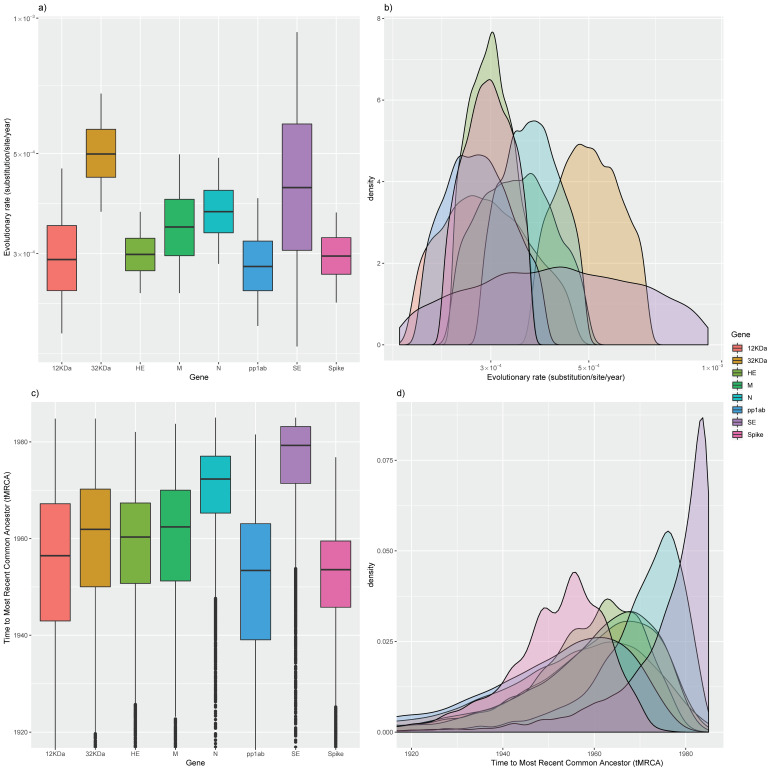
Upper figure: Boxplot (**a**) and density plot (**b**) of the evolutionary rate posterior probability. Lower figure: Boxplot (**c**) and density plot (**d**) of time to most recent common ancestor (tMRCA) posterior probability. Results are reported for all considered genes. The 95% high posterior density (95HPD) intervals are reported for both figures. The median values are displayed as a bold line in the boxplots.

BEAST analysis performed on each ORF revealed largely overlapping evolutionary rates (Table 1 and Figure 2). Only the 32 KDa and SE protein showed higher values. Similarly, the tMRCA was fully comparable among different genes (Table 1 and Figure 2).

### 3.2. Spike Protein

#### 3.2.1. Phylodynamic Analysis

A total of 219 sequences with known collection dates were included in the analysis. Of those, 133 belonged to the American–Asian group and 86 to the European one. The majority were collected from domestic bovine (i.e., 173), while the others were collected from wild or captive animals. Two sequences originated from human beings. The collection sites and/or associated symptomatology were known for 161 strains (115 enteric and 46 respiratory).

**Figure 3:**
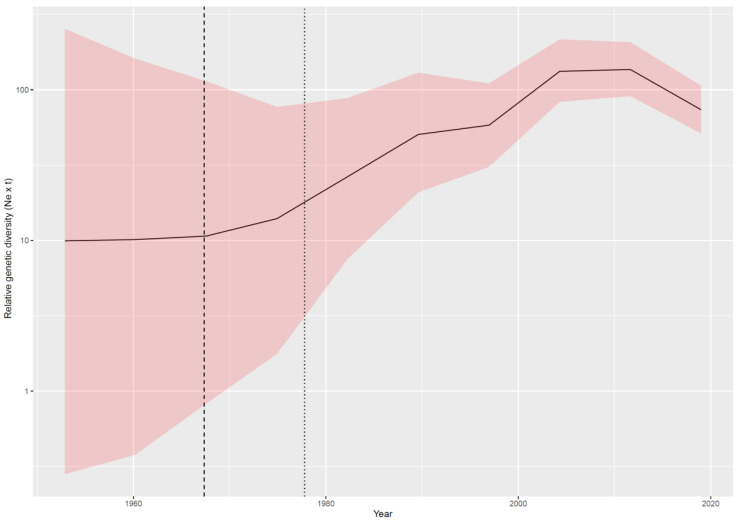
Relative genetic diversity (Ne x t) of the bovine coronavirus (BoCV) population over time. The mean value is reported in black, while the upper and lower 95HPD values are reported as a shaded area. The mean and upper 95HPD values of the time to Most Recent Common Ancestor (tMRCA) are reported as black dotted lines.

The evolutionary rate estimated using a broader S sequence dataset was slightly higher (i.e., mean = 8.39 × 10^−4^, median = 8.33 × 10^−4^, and 95HPD = 6.86 × 10^−4^ to 1.01 × 10^−3^) than that estimated on strains with a complete genome. Accordingly, the tMRCA was predicted in more recent times; however, the 95HPD intervals largely overlapped with previous estimates (mean = 1971.10, median = 1971.84, and 95HPD = 1959.89–1977.78). The reconstruction of BoCV population dynamics over time revealed a pattern featured by a progressive increase in relative genetic diversity until the most recent decades when a slight decrease was observed (Figure 3).

**Figure 4:**
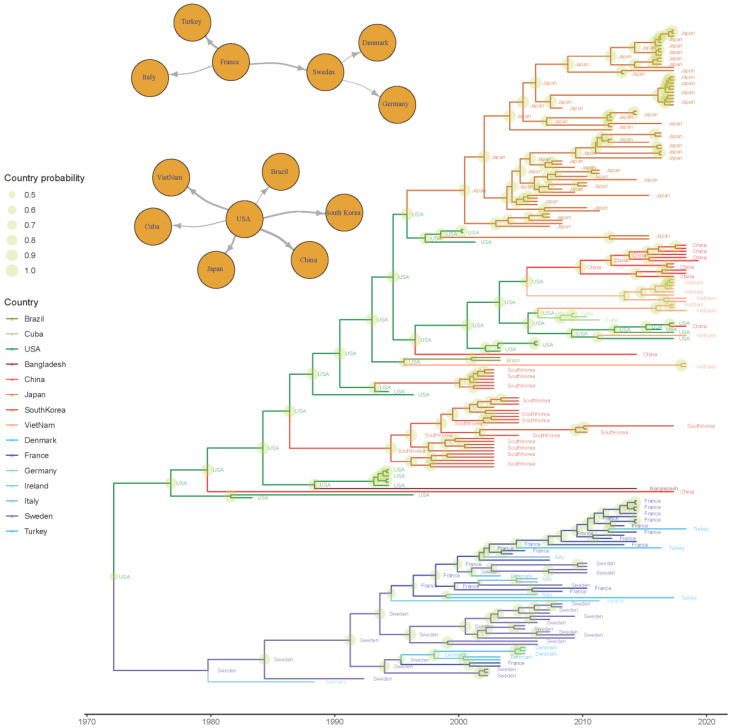
Time-scaled phylogenetic tree based on BoCV spike protein. The estimated ancestral geographic location has been color-coded (different nuances of green, red, and blue have been selected for American, Asian, and European countries), while the posterior probability is depicted as a circle on the corresponding node, whose size is proportional to the estimated value. The statistically supported migration routes have been represented in the upper left insert.

The phylogeographic analysis identified the US as the most likely origin of the virus, from where it might have spread to Sweden in the late 1970s and South Korea in the mid-1980s. However, the posterior probability associated with the origin (~0.6) and initial introduction site in Europe (~0.4) was low, thus preventing a precise reconstruction of the initial steps of viral dispersal. Thereafter, the two lineages followed independent pathways, with the strain initially introduced in Europe continuing to circulate in this continent (Figure 4 and Figure S1).

Particularly, between the 1980s and 1990s, the viral circulation first involved Germany (~1984), France (~1996), and Denmark (~1997) and then reached Ireland approximately in 2004. From France, it was introduced to Italy and Turkey (approximately in the late 1990s), likely through multiple introduction events (Figure 4).

**Figure 5:**
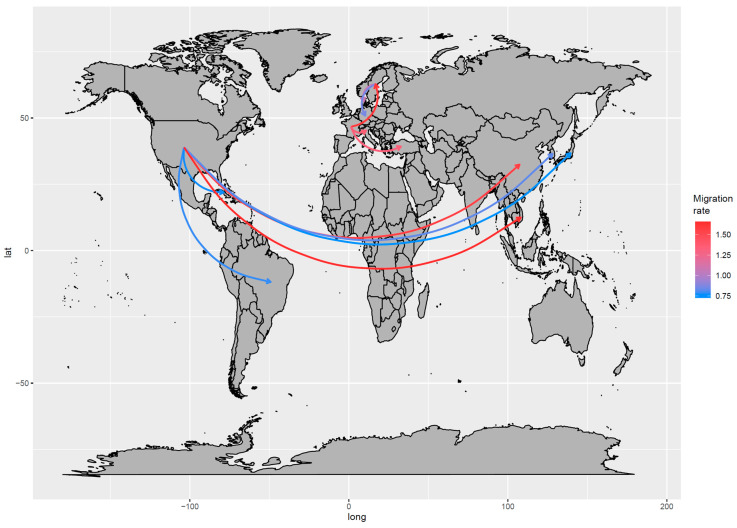
Well-supported migration paths between countries are depicted. The arrows indicate the directionality of the process, while the edge color is proportional to the base-10 logarithm of the migration rate. The location of each country has been matched with its centroid.

An intense connection was observed in the US, Asia, and Central–South America, with the US being the source of independent introduction of viral strains in different countries (Figure 4 and Figure S1), including South Korea (the 1980s), Brazil, China, Japan, Vietnam, and Bangladesh (1990s) and thereafter Cuba (~2005). The BSSVS allowed the identification of the statistically supported routes, confirming the above-mentioned scenario: The well-supported European and American–Asian migration rates formed two independent networks. While in the first network, Sweden and France seemed to share a significant role in the viral spreading, in the second network, the US was identified as the only source of BoCV introduction to other countries (Figure 4). Within Europe, a relatively high migration rate was observed, in contrast with the slow-spreading process from the US, with the viral introductions to China and Vietnam being the most remarkable exceptions characterized by multiple introduction events (Figure 4 and Figure 5).

**Figure 6:**
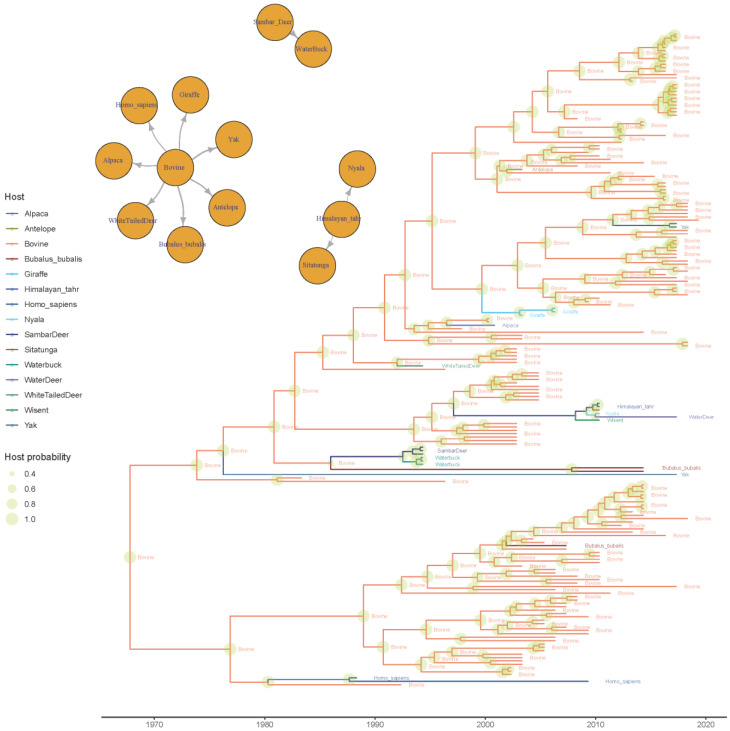
Time-scaled phylogenetic tree based on BoCV spike protein. The estimated ancestral host has been color-coded, while its posterior probability is depicted as a circle on the corresponding node, whose size is proportional to the estimated value. The statistically supported migration routes have been represented in the upper left insert.

Moreover, multiple host jumps were demonstrated to occur over time. Cattle were identified as the most relevant viral source for other domestic, captive, and wild species (Figure 6) and also the most likely origin of the BoCV-like strains sampled from human beings. Two additional independent contact networks were identified through BSSVS: the sambar deer as a viral source for the waterbuck, and the Himalayan tahr as a potential source of strains collected in nyalas and sitatungas.

#### 3.2.2. Selective Pressures

**Figure 7:**
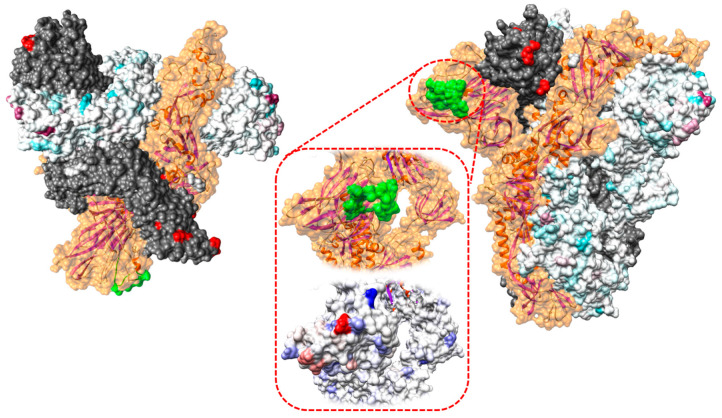
Upper (**left**) and lateral (**right**) view of the quaternary structure of the BoCV spike protein reconstructed using homology modeling. The appearance has been edited to highlight different selective pressure features. In the color-coded monomer, the difference between non-synonymous and synonymous substitution rates (dN–dS), calculated using FUBAR, is reported on the protein surface ranging from purple (dN > dS) to light blue (dN < dS). In the gray-colored monomer, sites under episodic diversifying selection are reported in red. In the remaining monomer, colored in orange, the receptor-binding domain (RBD) is highlighted in green. In the transparency, the ribbon structure is visualized. In the central insert, the RBD is magnified (upper image), and the strength of selective pressures (lower image) acting in that region is represented using the previously described scale. A more detailed representation of the overall protein structure is reported in the Animations (S1 and S2).

The analysis of the selective pressures acting on the S protein identified several sites under significant pervasive diversifying (Figure 7, Table 2, and Figure S2) and purifying selection using different methods (FEL, FUBAR, and SLAC). An even higher number of codons was detected under episodic diversifying selection with MEME (i.e., 4, 113, 155, 179, 220, 253, 257, 412, 499, 501, 509, 510, 546, 579, 717, 894, 1027, 1189, 1211, and 1298). The homology modeling of the spike quaternary structure allows us to display how the most intense diversifying pressures and most of those identified by MEME acted on the S surface (Figure 7 and Animation S1). While the receptor-binding domain (RBD) was largely under negative to neutral selection, nearby sites showed evidence of positive selection (Figure 7 and Animation S2).

Amino acid co-evolution analysis identified at least five pairs of amino acids whose mutations were linked with a posterior probability of >0.95 (Table 3 and Figure S3). Four sites were exposed on the S surface and in close proximity (Table 3), although amino acids 525–546 were on the opposite side of a protein protrusion. One of the exposed pairs included distant amino acids (244–531). Two relatively close amino acids (12 Å) embedded within the protein structure were also seen to co-evolve (Table 3 and Figure S3). aBSREL identified two branches only featured by a statistically significant episodic diversifying selection. Remarkably, those branches led to species different from cattle (i.e., bubalus and water deer).

Finally, the comparison of selective forces between strains collected from respiratory or enteric samples highlighted a significant episodic directional selective pressure pointing toward amino acids R, L, I, and E at positions 24, 690, 851, and 966 of respiratory strains, respectively (Figure S4).

## 4. Discussion

The recent emergence and spreading of SARS-CoV-2 have, once again, drawn attention towards coronaviruses and their evolution potential [39,40]. The present manuscript provides a 360° depiction of the epidemiological and evolutionary forces acting on BoCV. High mutation and recombination rates were demonstrated to cooperate in the genesis of the remarkable genotypic and phenotypic variability of coronaviruses and other viruses as well [41,42,43,44].

The analysis performed on the available whole genome sequences confirmed this tendency also for BoCV. Recombination appears as a frequent phenomenon, with 51% of sequences displaying evidence of potential recombination events. In silico recombination, detection must be critically evaluated, since several factors can affect its sensitivity and specificity [45,46]. Nevertheless, the combination of several methods, such as RDP4, GARD, and phylogenetic network analyses, strongly support the frequent occurrence of recombination. These pieces of evidence are in contrast with previous knowledge, which stated a marginal role of recombination in BoCV evolution [47]. However, the remarkable difference in the number of analyzed sequences and the use of complete genomes rather than individual genes or regions can easily justify the different results.

While no ORF or ORF region was preferentially affected by recombination events, some statistically significant recombination hot-spots were identified approximately in positions 4700 (pp1a), 21,600 (end of pp1ab/junction with region 32 KDa protein) and 23,900 (beginning of S) (based on the Mebus strain coding region). These results were also confirmed at the individual gene level and agree with the results reported by Salem et al. (2020) [9], who identified a potential recombination event within the S gene [9]. Interestingly, the presence of recombination hot-spots in the pp1ab and S gene has been described also in distantly related coronaviruses [48]. Since these regions are involved in replication efficiency and viral attachment, and thus in tropism and immunogenicity, the potential fitness gain featuring the new recombinant strains could favor the fixation, persistence, and spread of these genetic variants [49,50].

The other evolutive driving force of RNA viruses is their high mutation rate. Independently of the considered gene, the evolution rate was estimated to be approximately 10^−3^–10^−4^ substitutions/site/year, which is within the typical range of these viruses. Among the viral proteins, 32 KDa and SE were featured at a slightly higher rate. Although surprising, since these proteins are not the expected target of major selective pressures—i.e., immunity-induced proteins—it must be remembered that negative selection is the dominant force constraining and conditioning the viral phenotype and, indirectly, the viral genome. The less intense selective constraints acting on these proteins compared to major structural and non-structural proteins could allow greater flexibility and the accumulation of neutral and slightly deleterious mutations.

Since our initial aim was to compare the mutation rate of different genes, the estimation was performed on sequences obtained from complete genomes only to limit the potential biasing effect of using different datasets. When the broader complete S sequence dataset was analyzed, a slightly higher evolutionary rate was estimated. The inclusion of more sequences originating from a broader geographic area and encompassing a longer period likely provided a more representative depiction of the BoCV variability and evolution potential and thus more reliable estimations. Nevertheless, it must be stressed that the substitution rate’s order of magnitude was fully comparable and in agreement with previous studies [9,51], supporting the robustness of the overall obtained results.

As previously mentioned, selective pressure analysis performed on the broadest available S protein dataset demonstrated the dominant effect of purifying selection. However, different sites were detected under diversifying selection, particularly in the S1 region. Significantly, the even higher number of amino acid positions proved episodic diversifying selection, which supports, as demonstrated for other coronaviruses, the idea that evolution is not pervasive and largely acts through selective bursts [42], such as those induced by environmental changes, the invasion of new niches, etc. The homology modeling based on similar templates—and particularly on the closely related human HCoV-OC43—allowed the reliable reconstruction of its quaternary structure as the S protein is conserved [52]. The stronger selective diversifying pressures were clearly acting on the amino acids exposed on the S surface, allowing us to speculate on a major role for the host immune pressure on spike evolution. Interestingly, amino acids that form part of the receptor-binding domain were largely subject to negative to neutral selection, suggesting that the need to preserve the interaction with sialic acid prevented protein diversification, even in the presence of a likely immune pressure. This hypothesis is supported by the evidence that ligand-interacting residues or residues that are indirectly involved in the formation of the binding site are also strictly conserved in other species, such as HcoV-OC43, HCoV-HKU1, and porcine hemagglutinating encephalomyelitis virus (PHEV) [3,52]. Other amino acids near the receptor pockets were subject to diversifying selection. Therefore, the binding of antibodies to these epitopes could prevent viral attachment through sterical inhibition and consequently promote BoCV diversification in these less functionally constrained regions (Figure 7).

Protein function is the result of a long-lasting process of evolution involving the fine interaction of different domains. Therefore, after a mutation occurs at one site, compensatory mutations are often required for an actual fitness gain [53]. A compatible pattern was confirmed for BoCV. Despite a conservative 0.95 posterior probability threshold being selected in the BGM analysis, five sites were determined to co-evolve. Three of those were located on the viral surface in nearby positions, suggesting the role of linked mutations in the interaction of the S protein with the host. However, the identification of associated substitutions involving internal amino acids suggests the relevance of preserving the overall protein stability.

Besides immunity, host tropism is among the factors that could favor spike variation. The evaluation of host-jump patterns identified cattle as the principal source of BoCV introduction to other domestic, captive, and wild species, even through multiple introduction events, confirming its relevance as the main viral reservoir [10]. Interestingly, even the human cases of BoCV-like infection were traced back to a bovine source, confirming the previous hypothesis [54]. The origin of other human (HCoV-OC43) and animal coronaviruses was previously attributed to bovine species [55,56,57,58]. Therefore, the present results further show the zoonotic potential of these viruses and the need for further investigations on this topic.

The analysis of branch-specific selective pressure identified only two non-bovine lineages undergoing episodic selection during their history, which could suggest the actual occurrence of progressive host adaptation at the spike gene level. However, no clear association was identified between the host and amino acid composition in sites detected under selection by other methods, and none of the selected sites was directly involved in the receptor-binding domain (Figure 7) [52]. Unfortunately, only limited and extemporaneous transmission chains involving non-bovine species were investigated, thus impairing our ability to detect the long-term adaptation to new species.

Similar limitations were evident when the role of non-cattle species in maintaining infection and serving as an intermediate source for further host jumps was evaluated. Although statistically significant links were demonstrated both between cattle and wild species and among wild species, several of the latter strains were obtained from zoos or natural reserves, where species originating from different geographic areas are artificially kept in close contact. Therefore, while the susceptibility of these species and their ability to transmit infection was proven, their actual role in natural settings remains largely unknown. Overall, no strict evidence of an actual host-specific adaptation could be provided, and the detection of BoCV in such a plethora of different species [10] could be attributed to its innate plasticity.

A relatively similar picture was drawn from the evaluation of tissue tropism. Some reports suggested that all BCoV strains are similar at genomic and antigenic levels [4,59], while others proposed that enteric and respiratory strains are genetically and antigenically different [8,60]. The present study demonstrates that no long-term tissue adaptation has occurred, at least in the spike gene, without a clear association being demonstrated between tree topology and tropism. While certain small clusters were featured by a preferential tropism, they largely originated from the same study, introducing a dominant confounding effect of the involved animals, study location, and purpose. Other options remain available. First, mutations in other proteins or even non-coding regions, as proven for other viruses [61,62], would be worthy of investigation when a broader and less biased dataset of complete genomes becomes available. Another non-mutually-exclusive hypothesis is that specific mutations that occur in a limited site number could favor a change in viral tropism. Episodic directional selection analysis identified four sites pointing toward different preferential amino acids in respiratory strains. However, in this case, a critical evaluation of the statistically obtained results also seems to lessen their relevance. The number of strains affected by these mutations was limited, and although certain mutations were peculiar for respiratory strains, other genetically related viruses with the same tropism showed the amino acid featuring enteric strains (Figure S4). Consequently, even if an advantage in the specific tissue replications conferred by these mutations cannot be excluded, none of them seems to be necessary for effective tissue adaptation. Further experimental studies based on reverse genetics could be of great help in the evaluation of this still unsolved issue.

Finally, the BoCV dispersal pattern was evaluated; the actual origin of BoCV remains controversial since the ancient node ancestral states were featured by a low posterior probability. However, as previously proposed [1,9,63], compartmentalization between European and American–Asian strains was demonstrated and arose around the late 1970s to early 1980s. Europe is a market that is essentially closed to external cattle importations but featured by a dense internal livestock trade [64]. Accordingly, a high migration rate of viral strains could be estimated among European countries, similar to the situation described for other coronaviruses (and other viruses as well) of veterinary interest [65,66]. France and Sweden were identified as the main spreading sources. France, in particular, is the most important cattle exporter to several counties, including Italy, which is one of the major importers. This viral migration pattern is thus fully compatible with the importation of feedlot calves for the last growth and fattening phases when significant clinical problems and economic losses occur as a consequence of viral infection and shipping/acclimatization related stresses. Sweden, on the other hand, currently has a much lower impact on the bovine trade. Nevertheless, Swedish exportation was more prominent in the late 1960s and 1970s (FAOSTAT; www.fao.org/faostat/), when the initial BoCV spreading was estimated to occur. This evidence demonstrates that more intense monitoring and preventive measures, such as vaccination administration in young animals before international trade, could not only reduce the clinical impact but also the transferal and mixing of BoCV strains within Europe.

A comparable scenario, although at a much broader geographical level, was observed in the American–Asian group, where viral strain migration mimicked the livestock importation from the US to other countries. For example, in South Korea, where cattle trade from the US has been active since the 1960s, BoCV was imported approximately in the 1980s, while in Japan, where a huge increase in cattle importation was delayed by a decade, BoCV introduction was estimated in the 1990s (U.S. Department of Agriculture; https://www.usda.gov/). The Vietnamese and Cuban cases are even more illustrative. Due to politically-imposed restrictions, an active trade began only in the 1990s [67] and the middle 2000s [68] and was promptly followed by viral introduction. Even if the viral migration rate was overall less intense than within European borders, some exceptions were observed, particularly in Asian countries, where several independent introduction events from the same source (i.e., the US) were proven, testifying a largely unconstrained viral flux even over extremely long distances.

This spreading was paralleled by a progressive increase in viral population size that was ascribable to the progressive invasion of new niches characterized by relatively immunologically naïve populations. The slight decline observed in the last decades could be due to the increasing awareness of the clinical and economic relevance of this infection and to the implementation of more effective control measures, including planned vaccination protocols. However, an artifactual reduction cannot be excluded. In fact, because the sequences are not obtained from systematic studies, it is unavoidable that the most recent sequences originate from a limited number of countries. Therefore, the observed reduction could actually be representative of the analysis of a “more restricted” geographic area (and thus viral population) rather than of a worldwide decreasing pattern [69]. In the future, the accumulation of new sequences from a more representative geographical dataset would allow this doubt to be excluded. In fact, the most relevant room for improvement is represented by the relatively limited number of available sequences and by their uneven distribution among countries and species. Although the implemented methods are more resistant than traditional epidemiology to such a source of bias, their potential effect on parameter estimations, such as tMRCA, migration, and host jump patterns, cannot be excluded. Therefore, continuous efforts should be made in the acquisition and sharing of more sequences from previously neglected areas and ecological niches.

## 5. Conclusions

Overall, the present study provides a comprehensive depiction of evolutive and epidemiological BoCV pathways at a worldwide level. A remarkable role of recombination and high mutation rate has been demonstrated to generate the genetic variability on which selective pressures can act. Immune pressure is likely to play a major role, favoring the diversification of the spike protein in regions exposed on the viral surface. The occurrence of co-evolution linking different sites is likely the result of the compensatory mutation necessary to preserve protein stability and functionality. On the contrary, while some weak evidence of host/tissue tropism-induced selection could be detected, overall, BCoV tropism appears to be more closely linked to innate flexibility rather than to specific adaptations. However, the amount and structure of available sequences prevented definitive conclusions and should prompt more intensive sequencing and analysis activity.

## Figures and Tables

**Table 1:** Estimation of evolutionary rate and time to most recent common ancestor (tMRCA) for different protein-coding genes. Mean, median, and 95% high posterior probability (95HPD) summary statistics are reported.

Parameter	Protein	Mean	Median	Lower 95HDP	Higher 95HDP
**Evolutionary rate**	pp1ab	2.86 × 10^−4^	2.80 × 10^−4^	2.12 × 10^−4^	3.86 × 10^−4^
	32 KDa	5.06 × 10^−4^	4.98 × 10^−4^	3.81 × 10^−4^	6.61 × 10^−4^
	HE	3.00 × 10^−4^	2.98 × 10^−4^	2.49 × 10^−4^	3.64 × 10^−4^
	S	2.97 × 10^−4^	2.96 × 10^−4^	2.38 × 10^−4^	3.62 × 10^−4^
	12 KDa	3.01 × 10^−4^	2.90 × 10^−4^	2.4 × 10^−4^	4.42 × 10^−4^
	SE	4.55 × 10^−4^	4.20 × 10^−4^	2.00 × 10^−4^	8.65 × 10^−4^
	M	3.49 × 10^−4^	3.43 × 10^−4^	2.51 × 10^−4^	4.80 × 10^−4^
	N	3.75 × 10^−4^	3.71 × 10^−4^	2.90 × 10^−4^	4.78 × 10
**tMRCA**	pp1ab	1947.98	1953.40	1906.23	1971.93
	32 KDa	1957.34	1961.90	1921.92	1977.44
	HE	1956.83	1960.32	1926.97	1974.58
	S	1951.37	1953.57	1928.94	1966.89
	12 KDa	1952.13	1956.46	1912.25	1977.13
	SE	1974.07	1979.27	1947.92	1984.82
	M	1957.99	1962.41	1924.42	1977.13
	N	1969.51	1972.32	1947.62	1981.74

**Table 2:** Codons of the spike protein detected under statistically significant diversifying selection using different methods. The difference between non-synonymous and synonymous substitution rates (dN–dS) and respective significance level (*p*-value or posterior probability) are reported.

	FEL		FUBAR		SLAC	
Position	dN-dS	*p*-Value	dN-dS	Posterior Probability	dN-dS	*p*-Value
113	4.714	0.011	7.060	0.993	5.711	0.013
174	-	-	-	-	4.000	0.039
179	9.127	0.030	17.724	0.994	10.129477	0.012
257	3.009	0.039	4.547	0.937	-	-
400	-	-	3.326	0.912	-	-
499	5.817	0.013	12.690	0.997	5.816	0.021
501	7.518	0.001	14.566	1.000	12.412	0.000
509	9.840	0.001	17.373	1.000	9.071	0.002
510	3.790	0.024	6.358	0.984	4.722	0.030
525	-	-	8.060	0.917	6.462	0.034
572	-	-	4.962	0.938	-	-
579	3.456	0.032	5.916	0.972	4.219	0.046
966	-	-	7.742	0.972	-	-
1298	1.307	0.026	1.403	0.918	-	-

- Non-significant pressure with the specific method.

**Table 3:** Pairs of spike amino acids proven to co-evolve. For each pair, the position of the involved amino acids and their distances are reported.

Amino Acid 1	Amino Acid 2	Position Amino Acid 1	Position Amino Acid 2	Distance (Å)
121	260	Internal	Internal	12
146	148	External	External	5.4
147	151	External	External	6.8
244	531	External	External	29.1
525	546	External	External	12.9

## Supplementary Materials

The following materials are available online at https://www.mdpi.com/1999-4915/12/11/1285/s1, Figure S1: Migration of BoCV over time. Migration events are represented as arrows (chronologically color-coded from black to red) connecting the involved countries. The size of the polygons around a sampling location is proportional to the number of lineages maintaining the location. Figure S2: Depiction of selective pressures acting on spike codon positions estimated using different methods. Statistical significance is depicted by color-coded points. Figure S3: Depiction of the amino acid pairs detected to co-evolve. On the left, the amino acid pair detected to co-evolve is plotted against the phylogenetic tree. The tips of the tree have been color-coded according to the collection host. On the right, the involved amino acids are highlighted on the ribbon representation of the spike protein quaternary structure. The first amino acid is represented in red; the second is presented in blue. The spike surface is also displayed in the transparency and color-coded using the same scheme when the involved amino acids are exposed to the external environment. Lateral or top views have been selected depending on the particular pair to display the disposition of involved amino acids better. Figure S4: Depiction of codons under directional selection in relation to tissue tropism. The amino acid pair detected under directional selection is plotted against the phylogenetic tree. The tips of the tree have been color-coded according to viral tropism. Animation S1: Selective pressures acting on the spike protein. The appearance has been edited to highlight different selective pressure features. In the color-coded monomer, the dN–dS difference, calculated using FUBAR, is reported on the protein surface ranging from purple (dN > dS) to light blue (dN < dS). In the gray-colored monomer, sites under episodic diversifying selection are reported in red. In the last monomer, colored in orange, the receptor-binding domain (RBD) is highlighted in green. The ribbon structure is visualized in transparency. Animation S2. Focus of selective pressures acting on the receptor-binding domain (highlighted in green). The dN–dS difference, calculated using FUBAR, is reported on the protein surface ranging from red (dN > dS) to blue (dN < dS).
