# Supplementary material for: Bovine Coronavirus: Variability, Evolution, and Dispersal Patterns of a No Longer Neglected Betacoronavirus
Source: Viruses. 2020 Nov 10;12(11):1285. doi: 10.3390/v12111285 (PMC7697035; doi:10.3390/v12111285)
Supplement: Supplementary file 1 [file viruses-12-01285-s001.zip › Supplementary figure 3.pdf]

Supplementary figure 3

On the left, the amino acid pair detected to co-evolve is plotted against the phylogenetic tree. The tips of the tree have been color-coded according to the collection host. On the right, the involved amino acids are highlighted on the ribbon representation of the Spike protein quaternary structure. The first amino acid is represented in red, the second in blue. The Spike surface is also displayed in transparency and, when the involved amino acids are exposed on the external environment, color-coded using the same scheme. Lateral or top view have been selected depending on the particular pair to better display the disposition of involved amino acids.

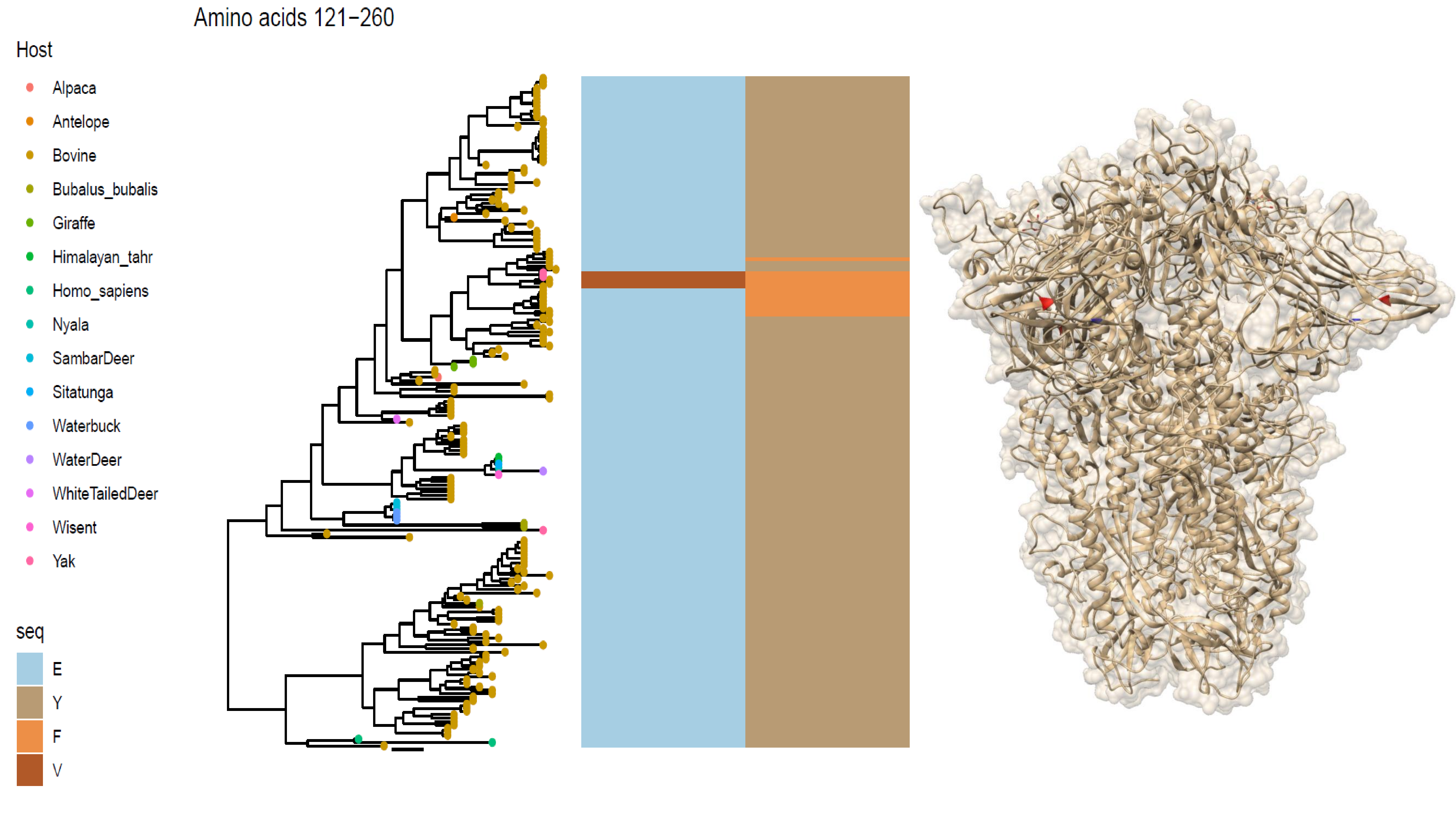
Amino acids 121−260
Host
Alpaca
Antelope
Bovine
Bubalus_bubalis
Giraffe
Himalayan_tahr
Homo_sapiens
Nyala
SambarDeer
Sitatunga
Waterbuck
WaterDeer
WhiteTailedDeer
Wisent
Yak
seq
E
Y
F
V

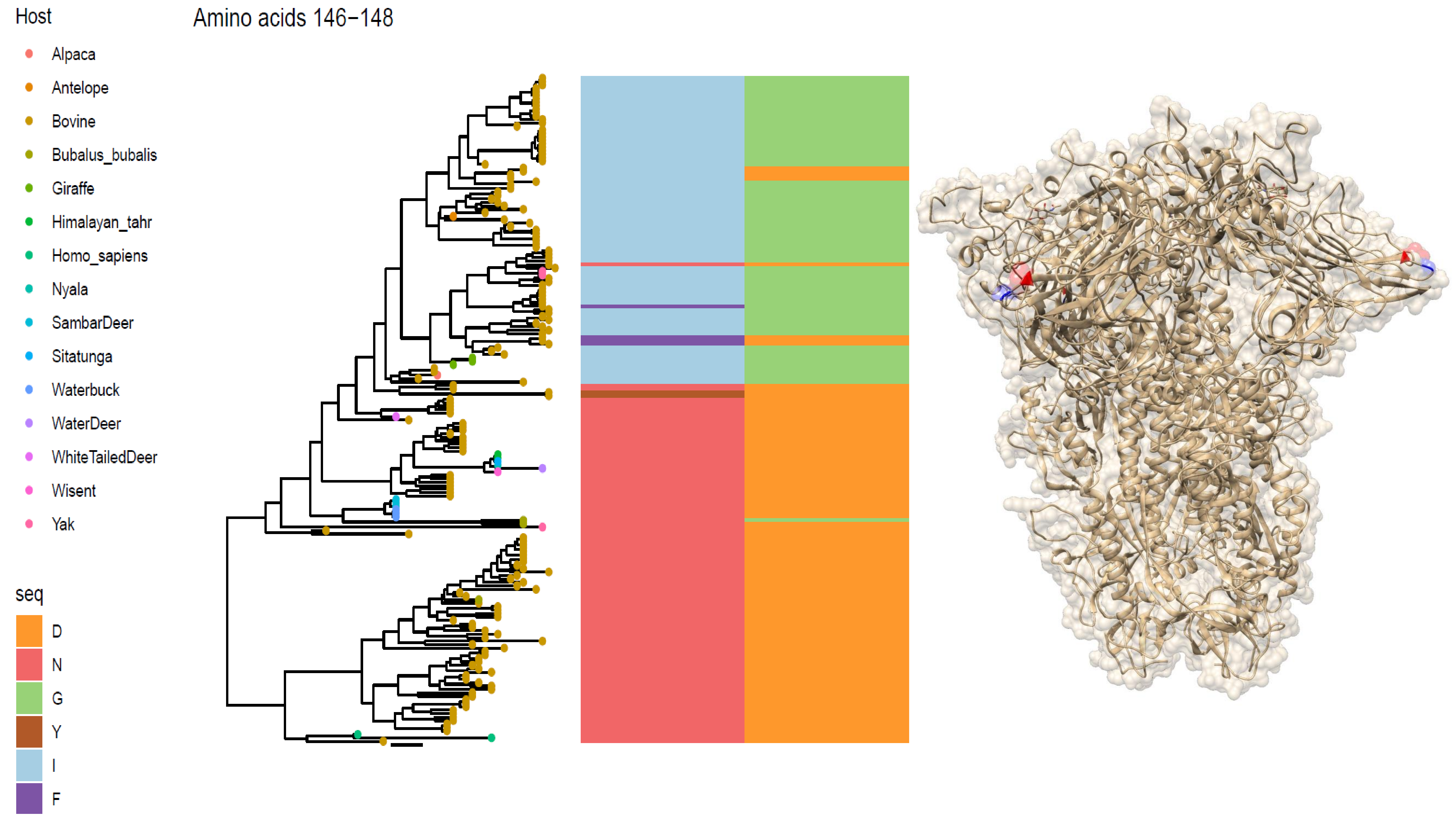
Host
Alpaca
Antelope
Bovine
Bubalus_bubalis
Giraffe
Himalayan_tahr
Homo_sapiens
Nyala
SambarDeer
Sitatunga
Waterbuck
WaterDeer
WhiteTailedDeer
Wisent
Yak
seq
D
N
G
Y
I
F
Amino acids 146−148

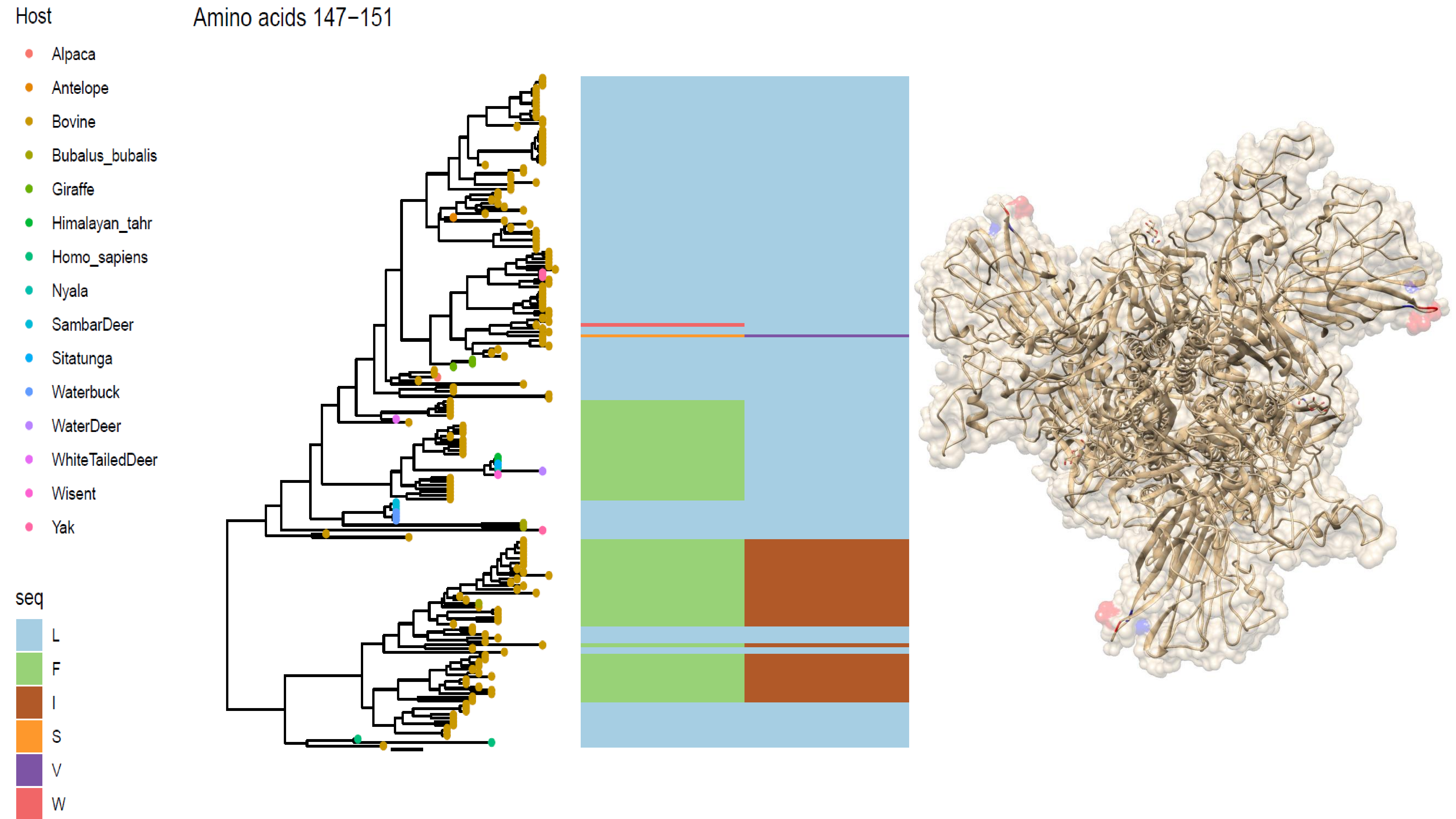
Host
Alpaca
Antelope
Bovine
Bubalus_bubalis
Giraffe
Himalayan_tahr
Homo_sapiens
Nyala
SambarDeer
Sitatunga
Waterbuck
WaterDeer
WhiteTailedDeer
Wisent
Yak
seq
L
F
I
S
V
W
Amino acids 147−151

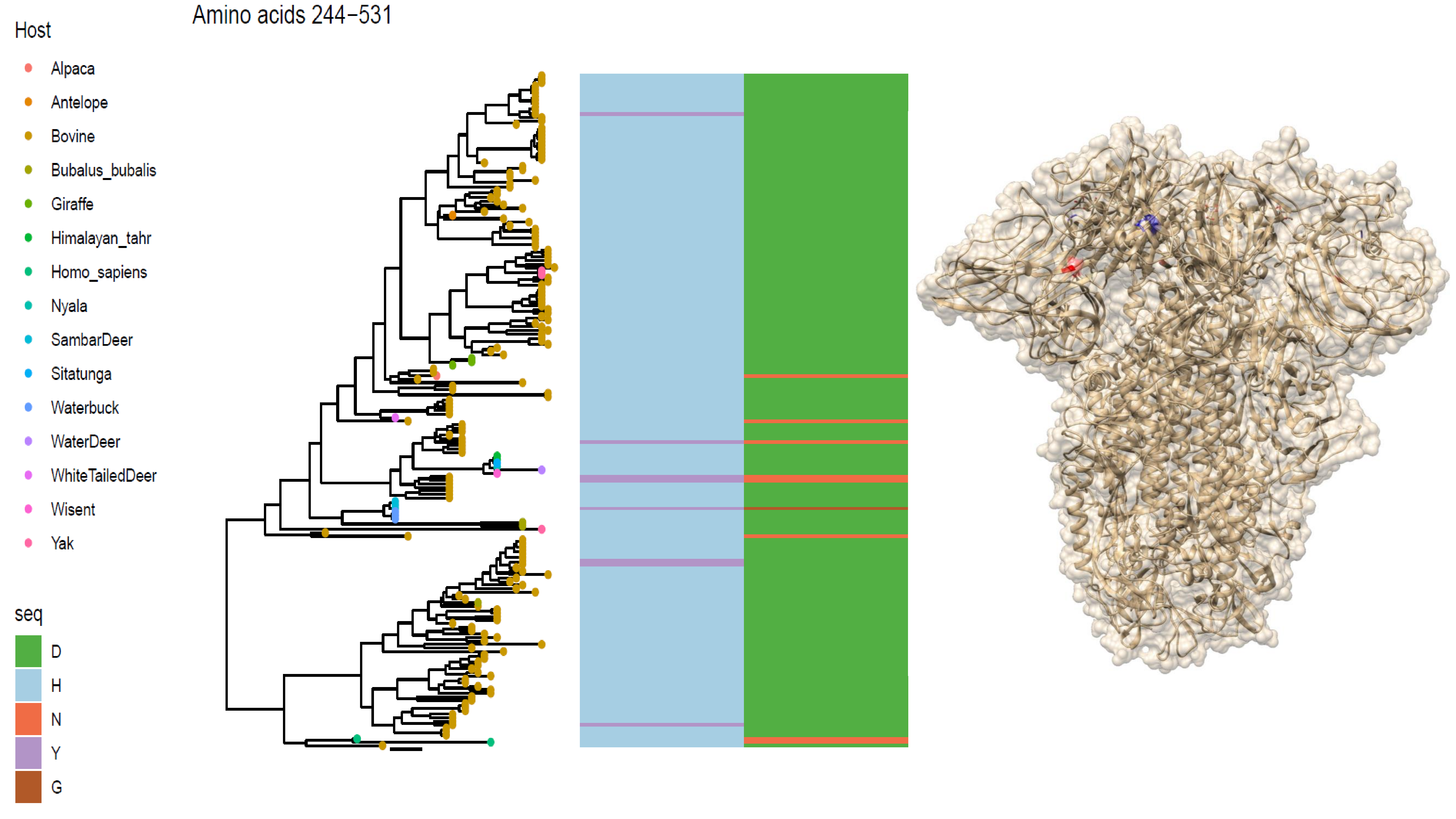
Amino acids 244-531
Host
Alpaca
Antelope
Bovine
Bubalus_bubalis
Giraffe
Himalayan_tahr
Homo_sapiens
Nyala
SambarDeer
Sitatunga
Waterbuck
WaterDeer
WhiteTailedDeer
Wisent
Yak
seq
D
H
N
Y
G

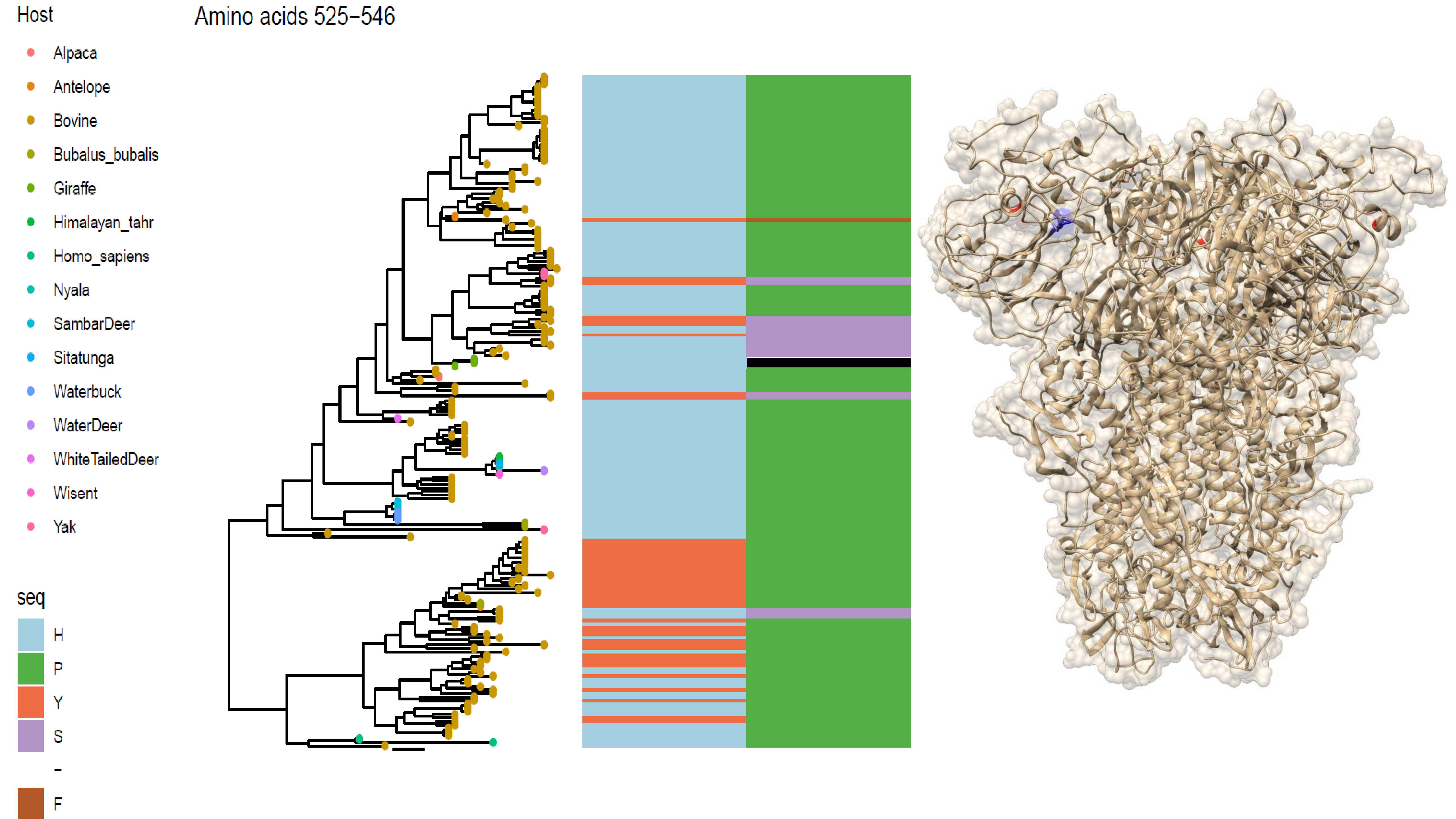
Host
Alpaca
Antelope
Bovine
Bubalus_bubalis
Giraffe
Himalayan_tahr
Homo_sapiens
Nyala
SambarDeer
Sitatunga
Waterbuck
WaterDeer
WhiteTailedDeer
Wisent
Yak
seq
H
P
Y
S
-
F
Amino acids 525−546
