# Supplementary figures and images for: Bovine Coronavirus: Variability, Evolution, and Dispersal Patterns of a No Longer Neglected Betacoronavirus
Source: Viruses. 2020 Nov 10;12(11):1285. doi: 10.3390/v12111285 (PMC7697035; doi:10.3390/v12111285)

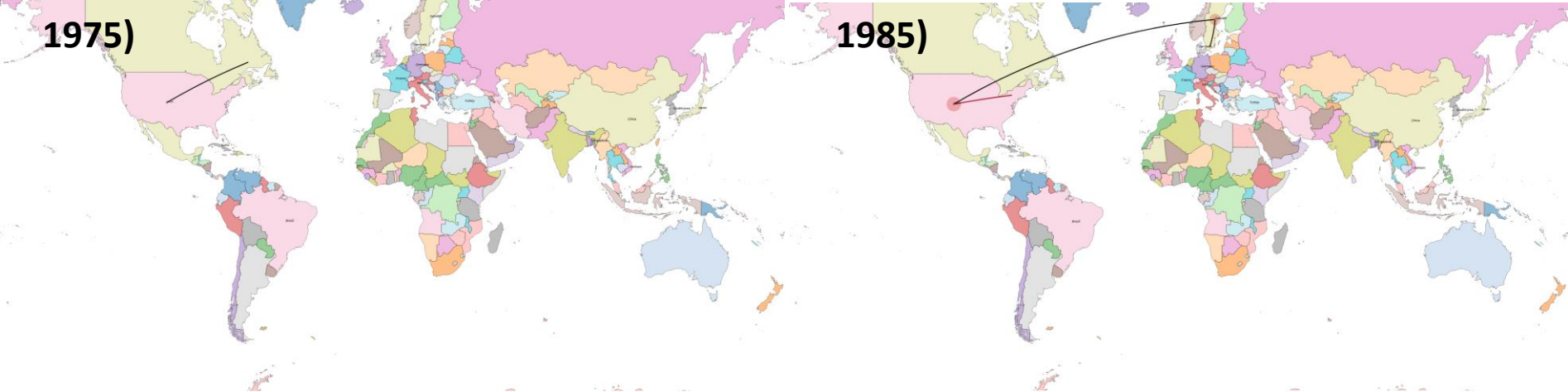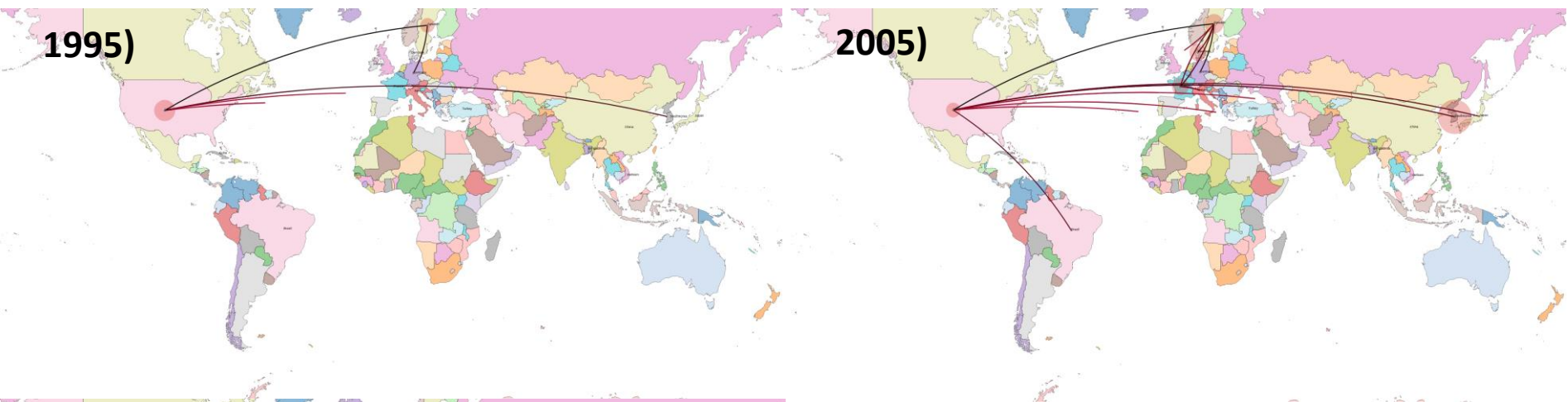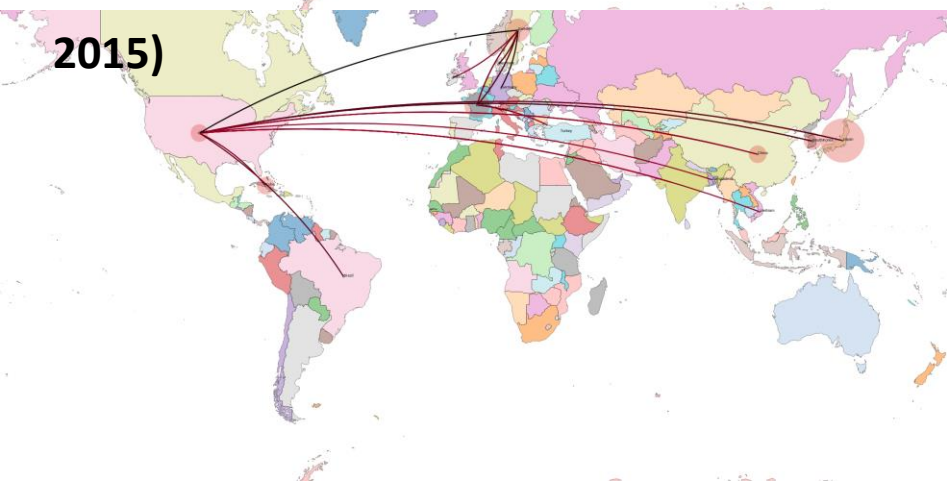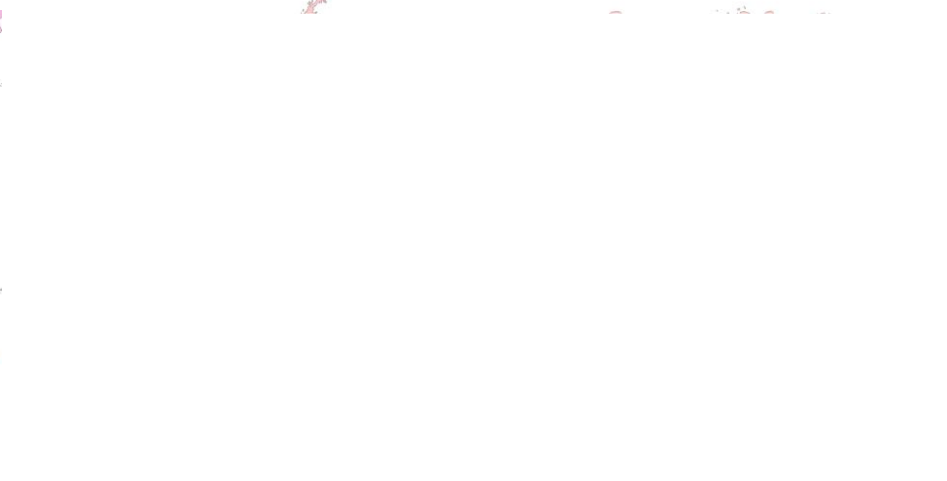

Supplement: Supplementary file 1 [file viruses-12-01285-s001.zip › Supplementary figure 1.pdf]

FEL

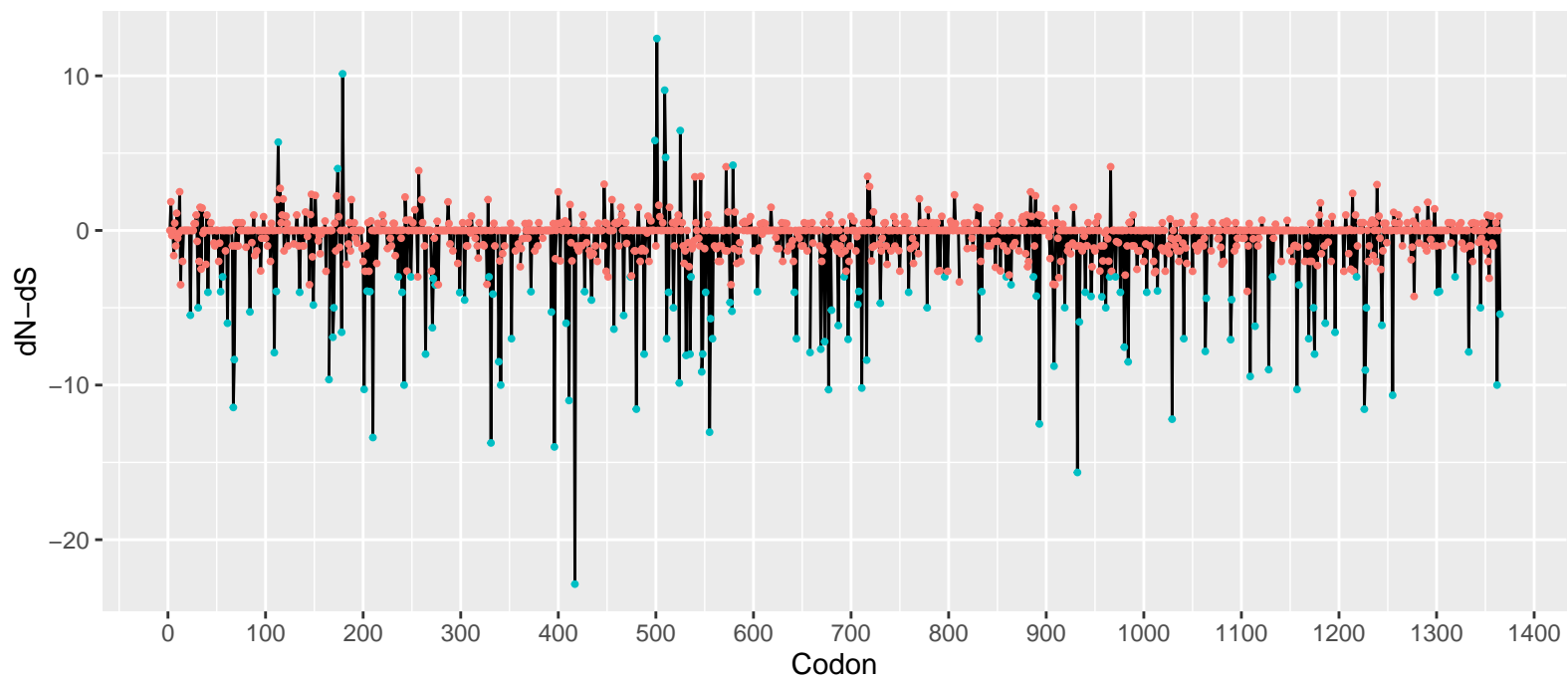

FUBAR

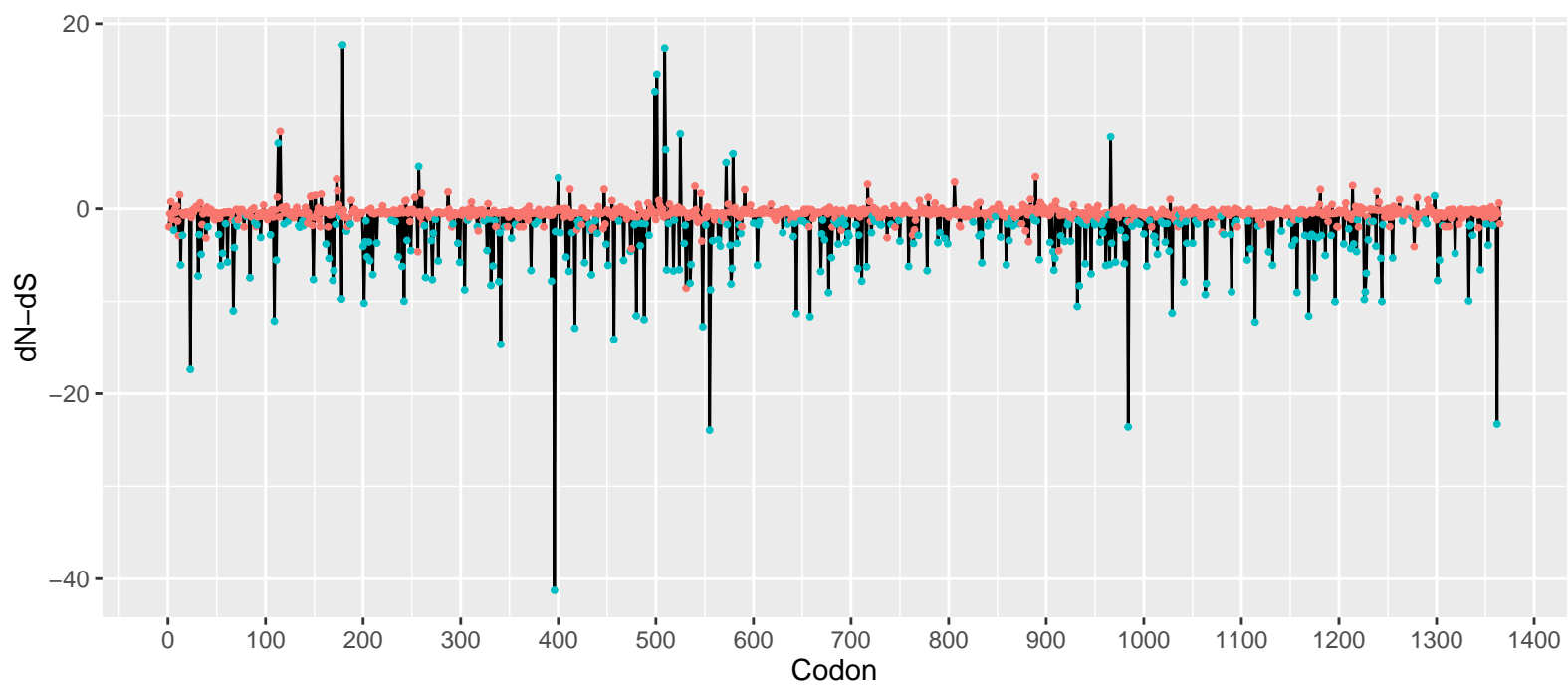

SLAC

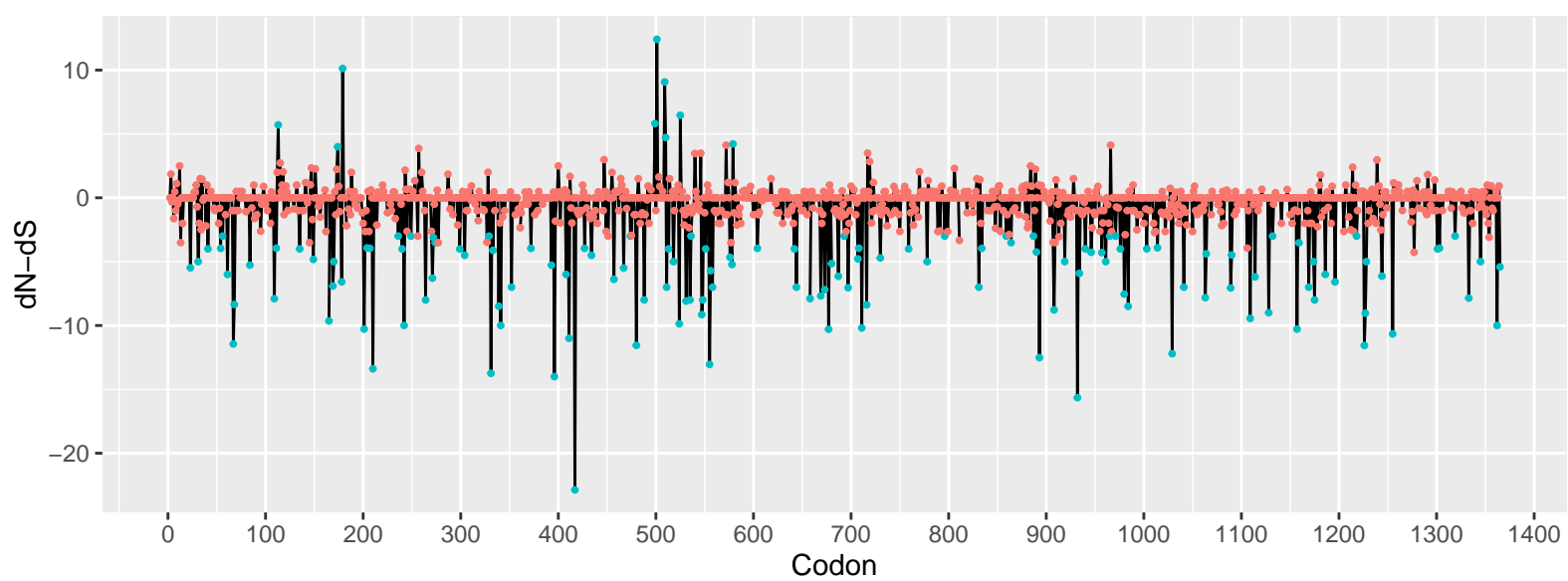

Statistical significance

• No • Yes

Supplement: Supplementary file 1 [file viruses-12-01285-s001.zip › Supplementary figure 2.pdf]

# Codons under Episodic Directional selection

## Amino acid

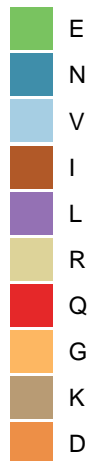

## Tropism

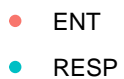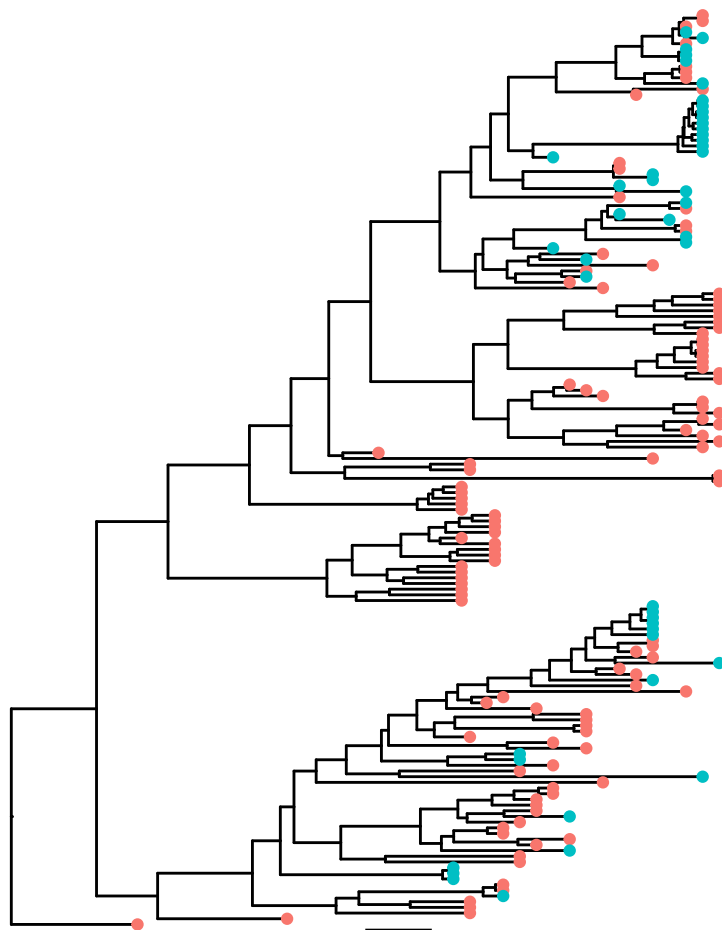

24 690 851 966

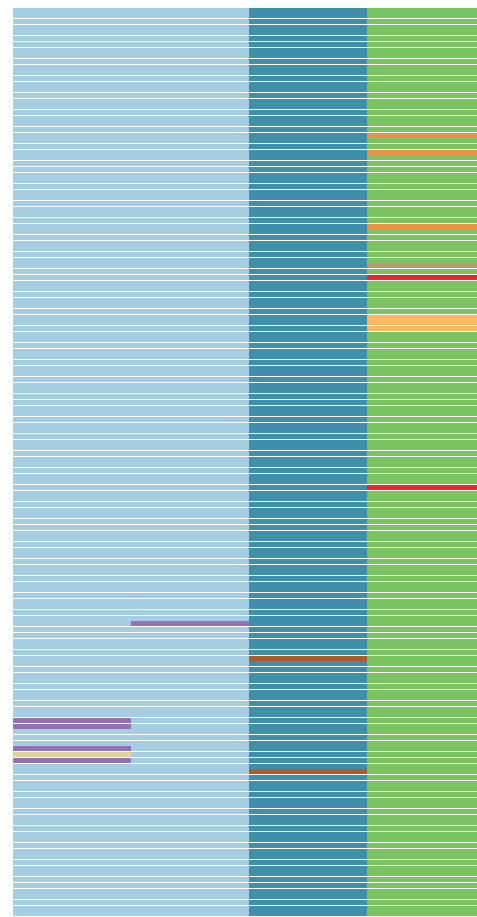

Supplement: Supplementary file 1 [file viruses-12-01285-s001.zip › Supplementary figure 4.pdf]
